# Electrode Mass Balancing as an Inexpensive and Simple Method to Increase the Capacitance of Electric Double-Layer Capacitors

**DOI:** 10.1371/journal.pone.0163146

**Published:** 2016-09-22

**Authors:** Britta Andres, Ann-Christine Engström, Nicklas Blomquist, Sven Forsberg, Christina Dahlström, Håkan Olin

**Affiliations:** 1 Department of Natural Sciences, Mid Sweden University, Sundsvall, Sweden; 2 STT Emtec AB, Sundsvall, Sweden; 3 Department of Chemical Engineering, Mid Sweden University, Sundsvall, Sweden; Monash University, AUSTRALIA

## Abstract

Symmetric electric double-layer capacitors (EDLCs) have equal masses of the same active material in both electrodes. However, having equal electrode masses may prevent the EDLC to have the largest possible specific capacitance if the sizes of the hydrated anions and cations in the electrolyte differ because the electrodes and the electrolyte may not be completely utilized. Here we demonstrate how this issue can be resolved by mass balancing. If the electrode masses are adjusted according to the size of the ions, one can easily increase an EDLC’s specific capacitance. To that end, we performed galvanostatic cycling to measure the capacitances of symmetric EDLCs with different electrode mass ratios using four aqueous electrolytes— Na_2_SO_4_, H_2_SO_4_, NaOH, and KOH (all with a concentration of 1 M)—and compared these to the theoretical optimal electrode mass ratio that we calculated using the sizes of the hydrated ions. Both the theoretical and experimental values revealed lower-than-1 optimal electrode ratios for all electrolytes except KOH. The largest increase in capacitance was obtained for EDLCs with NaOH as electrolyte. Specifically, we demonstrate an increase of the specific capacitance by 8.6% by adjusting the electrode mass ratio from 1 to 0.86. Our findings demonstrate that electrode mass balancing is a simple and inexpensive method to increase the capacitance of EDLCs. Furthermore, our results imply that one can reduce the amount of unused material in EDLCs and thus decrease their weight, volume and cost.

## Introduction

Different methods to enhance the performance of supercapacitors have been recently reported. New advanced and highly porous electrode materials [1, 2] have been developed, and functionalized electrode surfaces have been employed in supercapacitors [3]. The importance of matching electrolytes and electrode materials has been discussed, and Chmiola et al. [2, 4, 5] and Largeot et al. [6] have reported the importance of matching the electrode pore size to the size of the electrolyte ions or vice versa. These adjustments result in enhanced device performance. However, one must consider that most high-performance supercapacitors contain expensive and toxic materials. Thus, simple methods to improve a supercapacitor’s performance are desired when the choice of materials is limited due to factors such as cost savings, the environmental impact of the materials, or their availability.

In this study, we present a simple and effective approach to increase the capacitance of electric double-layer capacitors (EDLCs). Depending on the applied electrolyte, one should adjust the weight of the electrodes according to the size of the electrolyte ions to fully utilize the surface of both electrodes. The mass balancing will result in an increased capacitance.

### Perspective and purpose of this study

The main objective of this study is to produce and optimize inexpensive and environmentally friendly EDLCs for the automotive industry. One of today’s largest challenges of the automotive industry is the transition from the traditional internal combustion engine using fossil fuels to electric drives. Several factors still hinder this development. In addition to limited driving ranges and the poor infrastructure of charging stations in many regions, the high price of energy storage devices limits the commercialization of electric vehicles. Different types of energy storage devices, such as batteries and fuel cells, have been tested in electric vehicles. Supercapacitors are often proposed as intermediate power storage in combination with batteries, fuel cells or other long-term energy storage systems. Due to their high power density, supercapacitors can complement and protect electrochemical energy storage devices.

The EDLCs designed in this study are developed to be used as temporary energy storage devices in kinetic energy recovery systems (KERSs) in cars. Via a generator, the braking energy of a car can be converted into electrical energy, which can be stored in an EDLC. The stored energy can be used to accelerate the car using an electric motor or to power its on-board systems. Due to strict manufacturer demands on the weight, volume and price of supercapacitors, we focused on inexpensive materials, which are sufficient for this application. Although materials that offer a better performance in EDLCs are available, they do not meet the demands reviewed above. Furthermore, EDLCs should be maintenance free, reliable and highly efficient. In addition to these criteria, we aim to produce environmentally friendly and recyclable EDLCs.

### Mass-balancing principle

Symmetric EDLCs are composed of electrodes that contain the same active material in both the positive electrode and the negative electrode and use equal masses of active material in both electrodes. However, this electrode mass ratio might not be the optimal choice unless the electrolyte anions and cations have the same size. If the size of the electrolyte ions differs, one of the electrodes might not be fully covered with ions; thus, it may not be completely utilized. By contrast, there might be an excess of ions that do not contribute to the overall capacitance of an EDLC. In the case of smaller ions, the oppositely charged electrode is not completely covered with the smaller ions, whereas the other electrode is fully covered with the larger ions. If both electrodes are fully covered, there will be an excess of larger ions. These ions will not contribute to the capacitance.

To effectively use the entire surface area of both electrodes and to avoid excess electrolyte, the electrode mass ratio should be adjusted to the ion size ratio. The performance optimization of supercapacitors by mass balancing is widely used in asymmetric supercapacitors, e.g., hybrid supercapacitors, in which different active materials are used for the positive and negative electrodes [7–10]. Due to the differing electrode properties, such as capacitance and potential range, the electrode masses need to be balanced. Electrode mass balancing is employed to widen the voltage window of hybrid supercapacitors and pseudocapacitors, although it can also be applied for EDLCs [7–13]. Furthermore, a larger voltage window results in an increased energy density.

### Experimental setup and choice of materials

We investigated the optimal electrode mass ratio in different aqueous electrolytes. Aqueous electrolytes are an attractive alternative to organic electrolytes that are commonly used in supercapacitors. Most commercial supercapacitors use expensive and toxic electrolytes, such as tetraethylammonium tetrafluoroborate (TEA BF_4_) in acetonitrile [14]. However, only aqueous electrolytes qualify for application in inexpensive and environmentally friendly EDLCs. They offer many advantages, such as good ionic conductivity, low toxicity, non-flammability and low cost. Furthermore, supercapacitors with aqueous electrolytes offer higher capacitances and higher power densities than organic electrolytes. The following electrolytes were tested: sodium sulfate (Na_2_SO_4_), sulfuric acid (H_2_SO_4_), sodium hydroxide (NaOH) and potassium hydroxide (KOH). Potassium hydroxide and sulfuric acid were chosen because they are the most common aqueous electrolytes used in EDLCs [14]. Sodium sulfate was tested because it has an almost neutral pH, which is favorable for some electrode materials. Sodium hydroxide was investigated to complement the test results of sodium sulfate and potassium hydroxide. By choosing another anion or cation instead of the sulfate ion and the potassium ion, respectively, we expected to observe the influence of the ions.

The electrode material was a mixture of graphite and activated carbon with cellulose nanofibers (CNFs), also termed cellulose nanofibrils or nanofibrillated cellulose, as the binder. CNFs were used as the binder because they are an environmentally friendly alternative to fluorinated polymers, such as polytetrafluoroethylene (PTFE), that are commonly used in supercapacitors [14, 15]. Moreover, CNFs have previously demonstrated good mechanical and electrical properties as a binder in graphite electrodes [16]. An initial study showed that the addition of 10% CNFs, in relation to the amount of active material, exhibited the best performance in the tested composite. Graphite and activated carbon were chosen as the active material for the electrodes. Initial tests of these materials showed that a composite with 50% graphite and 50% activated carbon achieved both a good electrical conductivity and a favorable capacitance. Usually, the main component in EDLC electrodes is activated carbon with a high surface area, which provides a high capacitance. Increasing the amount of activated carbon, however, results in a reduced electric conductivity and a large resistance. Previously, we reported that the graphite used in this study exhibits a low sheet resistance and low electrical resistivity [16, 17]. These conditions are favorable for achieving a high power density. For the application of EDLCs in KERSs, a high power density is preferred over a high capacitance. Furthermore, EDLCs constructed from this graphite exhibit a good capacitance, cyclability and efficiency [16]. Thus, composites with equal amounts of graphite and activated carbon and an additional 10% CNFs were used for the electrodes in this study.

Supercapacitors with different electrode mass ratios were prepared, and galvanostatic cycling was performed to evaluate its influence on the capacitance of the EDLCs. Here, the electrode mass ratio is defined as the mass of the positive electrode divided by the mass of the negative electrode; see Eq (3). Galvanostatic cycling was chosen as the test procedure because it is a fast and simple method to determine the capacitance of supercapacitors. A two-electrode setup was used to imitate realistic operating conditions. By contrast to three-electrode methods, such as cyclic voltammetry, two-electrode galvanostatic cycling measures the entire cell instead of the performance of a single electrode. This approach is advantageous because it enables a quick test of a supercapacitor’s capacitance. The proposed mass-balancing method is a simple and relatively general method to optimize the capacitance of EDLCs. Although mass balancing may not achieve the highest possible capacitance, it can easily improve the resulting value. Exploiting a material’s full potential requires a time-consuming analysis of the components. Because this is not the aim of this study, we did not use any advanced electrochemical characterization methods.

## Materials and Methods

### Materials

The electrode materials, graphite (ABG 2025, SO# 5-42-25, from Superior Graphite) and activated carbon (Pulsorb 208CP Powder, from Chemviron Carbon), were used as received. Sulfuric acid (analytical grade, from VWR), sodium hydroxide (analytical grade, from VWR), potassium hydroxide (from Fluka) and sodium sulfate (analytical grade, from Merck) were diluted with deionized water to produce electrolytes with a concentration of 1. Greaseproof paper (untreated, grammage: 45 g/m^2^, from Nordic Paper) was employed as a separator and was used as received.

### Preparation of TEMPO-oxidized cellulose nanofibers

The process of the TEMPO-mediated oxidization of the CNFs followed the method described by Saito et al. [18]. We used 100 fully bleached softwood Kraft pulp. The pulp was diluted in 10 deionized water to 2.5% consistency. Then, 2 sodium bromide (NaBr, from Merck Millipore) per g dry pulp and 0.2 mmol/g TEMPO (2,2,6,6-tetramethyl-1-piperidinyloxy, from Sigma-Aldrich) was added to the pulp suspension. The suspension was mixed, and 10 sodium hypochlorite (NaClO, 14%, from VWR) per g dry pulp was added during stirring. To avoid large pH variations, small amounts of NaClO were added. The pH of the pulp suspension was adjusted to 9.5 by adding 1 M NaOH. The addition of NaClO and the subsequent pH adjustment were performed for 2 to 3 hours. Afterwards, the pulp was dewatered and thoroughly washed. The washed pulp was diluted to 1% consistency with deionized water. Approximately 700 ml of the pulp suspension was dispersed with the IKA T 25 digital Ultra-Turrax disperser (rotor: S25N-25F) at 15000 rpm for 30 minutes for proper mixing. This rotational speed corresponds to a shear velocity (circumferential speed) of 14.14m/s.

### Calculation of ion size ratio and electrode mass ratio

The following assumptions were made to develop a simplified model of the optimal electrode mass ratio. The objective of this study is to determine the optimal electrode mass ratio in order to increase the EDLC capacitance. However, we do not seek to fine-tune the mass ratio or the capacitance. Thus, a simplified model is sufficient for our purpose.

The ion size ratio and the theoretical electrode mass ratio were calculated from the size of the hydrated electrolyte ions. A table showing the radii of various hydrated ions can be found in [19]. Table 1 is an extract of this table and lists the ions tested in this study.

**Table 1:** Radii *r* of hydrated ions [19].

	ion	*r*/nm
cations	H^+^	0.282
	Na^+^	0.358
	K^+^	0.331
anions	OH^−^	0.300
	SO_4_^2−^	0.379

For an electrolyte molecule with the chemical formula *A*_*x*_ *B*_*y*_, the ion size ratio is calculated according to

$$\text{ion}\;\text{size}\;\text{ratio}=\frac{x\cdot r_{A}}{y\cdot r_{B}}\;,$$ (1)

where *x* is the number of ion *A* in the molecule, *y* is the number of ion *B* in the molecule, *r*_*A*_ is the radius of the hydrated ion *A*, and *r*_*B*_ is the radius of the hydrated ion *B*. The theoretical electrode mass ratio is the inverse of the ion size ratio:

$$\text{theoretical}\;\text{electrode}\;\text{mass}\;\text{ratio}=\frac{y\cdot r_{B}}{x\cdot r_{A}}\;.$$ (2)

The electrode mass ratio of the tested EDLCs was calculated by

$$\text{electrode}\;\text{mass}\;\text{ratio}=\frac{m_{+}}{m_{-}}\;,$$ (3)

where *m*_+_ is the mass of active material in the positive electrode, and *m*_−_ is the mass of active material in the negative electrode.

The largest increase in capacitance can be expected for electrolytes with a significant difference in ion size, i. e., a large anion and a much smaller cation (or vice versa). Moreover, one has to consider the valence of the ions because it determines the number of anions and cations that can form the electric double layer at the electrode-electrolyte interfaces. We expect the largest enhancement for sodium sulfate, which has a theoretical electrode mass ratio of 0.53, when considering the valence of the ions.

In general, electrolytes with small ions achieve larger capacitances than electrolytes composed of large ions at a given electrode size and weight. This result can be explained by a higher charge density at the electrode surface. Thus, the size of the electrolyte ions is crucial to produce EDLCs with large specific capacitances. Moreover, the shape of the hydrated ions should be considered because it influences the formation of the electric double layer.

### Preparation of electrodes

All of the electrodes were composed of the same composite. To prepare the electrodes, equal amounts of graphite and activated carbon were mixed with an additional 10% TEMPO-oxidized CNFs (10% of the total mass of graphite and activated carbon). Samples with a total weight of active material between 0.2 g and 0.7 g were prepared. Approximately 40 of deionized water was added to each mixture. The suspensions were dispersed for 10 minutes at 12000 rpm using the Ultra-Turrax disperser (rotor: S25N-10G). This rotational speed corresponds to a shear velocity (circumferential speed) of 4.71 m/s. Subsequently, the dispersions were filtrated on Millipore Durapore Membrane Filters (filter type: 0.22 GV, diameter: 90 mm) using a vacuum filtration funnel. Films with coating weights between 44 g/m^2^ and 154 g/m^2^ were obtained. All filters were dried at room temperature and cut into 3 cm × 3 squares.

### Characterization of electrode materials

The specific surface areas (SSAs) of the raw materials, graphite, activated carbon, and the composite were analyzed. Measurements according to the Brunauer-Emmett-Teller (BET) theory were performed using a Micromeritics Gemini 2370 BET instrument. Furthermore, digital images of the composite surface were obtained using a field emission scanning electron microscope (FESEM), Zeiss Merlin FESEM. Secondary electron images (SEIs) were generated using a 5 kV accelerating voltage and an in-lens detector.

### Assembly and testing of electric double-layer capacitors

The EDLCs were assembled by stacking electrodes and a separator in a test cell. The test cell is described in detail elsewhere [16]. Greaseproof paper was used as the separator between the electrodes. The components were wetted with electrolyte prior to assembling the test cell. EDLCs with different electrode mass ratios were prepared, and galvanostatic cycling was performed by using a LabVIEW-based PXI system. The EDLCs were cycled for 24 hours between 0 and 1V with a charge and discharge current of 8. The resulting discharge times and thus the amount of cycles per measurement depend on the EDLC capacitance and the applied current; see Eq (4). We selected a low current to allow comparison of devices with a high capacitance to EDLCs exhibiting a low capacitance. This low current resulted in discharge times between 150 and 665s giving 65 to 288 cycles per 24-hour measurement. We chose a test duration of 24 hours to evaluate the conditioning, efficiency and cycling stability of the EDLCs. To verify the results and eliminate the influence of the electrode thickness, five EDLCs with the optimal electrode mass ratio but varying electrode mass loadings were tested for each electrolyte. No significant variations were observed. The mean value of the specific capacitances was calculated. Furthermore, measurements of the EDLCs with specific capacitances close to the maximum were repeated by testing new EDLCs with the same electrode mass ratio. No significant variations were observed for the replicates.

#### Capacitance, specific capacitance and efficiency

Galvanostatic cycling was performed to evaluate the capacitance of the EDLCs. The capacitance *C* was calculated from the discharge curves according to

$$C=I\cdot \frac{dt}{dV}\;,$$ (4)

where *I* is the discharge current, d*t* is the discharge time, and d*V* is the cell potential difference. The specific capacitance *C*_*sp*_ was calculated by

$$C_{\text{sp}}=4\cdot \frac{C}{m}\;,$$ (5)

where *C* is the capacitance of the EDLC and *m* is the mass of the active material in both electrodes. The factor of 4 adjusts the cell capacitance *C* to the mass and capacitance of one electrode, considering that each EDLC consists of two capacitors, one on each electrode. In the case of our electrodes, only graphite and activated carbon were counted as active materials. CNFs were not considered as an active electrode material.

The efficiency of the EDLCs was calculated from the galvanostatic cycling results by dividing the discharge time by the charge time.

## Results

### Characterization of electrode materials

#### Specific surface area

The specific surface area of the electrode components graphite, activated carbon, and the produced composite was obtained by BET measurements. The results of these measurements are displayed in Table 2. As expected, the activated carbon exhibited a high specific surface area, whereas graphite had a low specific surface area. The composite, which contains equal amounts of activated carbon and graphite and an additional 10% CNFs, exhibited an intermediate specific surface area of 418 m^2^/g.

**Table 2:** Specific surface area (SSA) of electrode materials.

material	SSA/m^2^ g^−1^
activated carbon	903
graphite	19.8
composite	418

#### Surface structure

**Fig 1:**
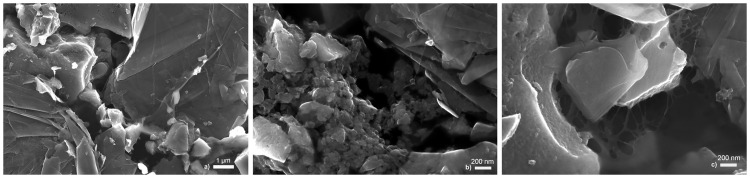
Structure of the composite surface taken with a field emission scanning electron microscope. The layered structure of the graphite flakes, the spherical shape of the activated carbon, and the cellulose nanofiber network are shown. a) magnification of 25000 ×; b) and c) magnification of 100000 ×.

The surface of the composite film was investigated using an FESEM. Fig 1 shows three images of different areas of the composite surface. Fig 1a and 1b show the layered graphite flakes, the spherical activated carbon and the network of CNFs. Fig 1c shows a close-up image of the nanofiber network. One can observe that the CNFs form a network that connects the graphite flakes and the activated carbon particles. This observation confirms the suitability of CNFs as a binder for graphite and activated carbon electrodes.

### Optimal electrode mass ratio

**Fig 2:**
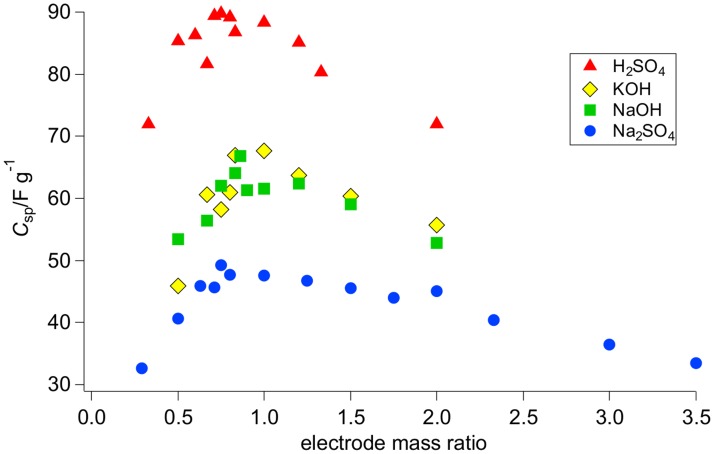
Influence of electrode mass ratio on specific capacitance of supercapacitors with different aqueous electrolytes.

Fig 2 shows the results of the capacitance measurements. Depending on the electrolyte used, we could detect an influence of the electrode mass ratio on the specific capacitance of the EDLCs. For each electrolyte, an optimal electrode mass ratio could be extracted from the maxima of the displayed curves; see Table 3. If more or less active material was used in either the positive or the negative electrode, only lower specific capacitances could be achieved. In addition, we detected large differences in the specific capacitance when comparing the different electrolytes. EDLCs with sulfuric acid as the electrolyte exhibited much higher capacitances than devices operating with one of the other electrolytes; see Fig 2 and Table 3. This finding can be explained by the small size and high mobility of the hydrogen cations. The advantage of small ions is that they can easily enter and fill small pores, which results in a higher capacitance. Large ions might not be able to enter narrow pores, leading to lower capacitances. Sodium sulfate exhibited the lowest specific capacitance of all the tested electrolytes. Sodium hydroxide and potassium hydroxide produced moderate specific capacitances with similar values for most tested electrode mass ratios. This result was expected because of the similar radii of hydrated potassium and sodium ions.

**Table 3:** Theoretical optimal electrode mass ratio emr_th_, measured optimal electrode mass ratio emr_m_, highest specific capacitance *C*_sp_, and specific capacitance increase *C*_incr_ of different aqueous electrolytes.

electrolyte	emr_th_	emr_m_	*C*_sp_/F g^−1^	*C*_incr_/%
Na_2_SO_4_	0.53	0.75	49.3	3.7
H_2_SO_4_	0.67	0.75	89.7	1.5
NaOH	0.84	0.86	66.8	8.6
KOH	0.91	1	67.6	0

Sulfuric acid and sodium sulfate exhibited the highest specific capacitance at an electrode mass ratio of 0.75. For sodium hydroxide, the highest specific capacitance was detected at an electrode mass ratio of 0.86. Potassium hydroxide, however, exhibited its largest specific capacitance with supercapacitors having equal electrode masses. An optimal electrode mass ratio equal to 1 or slightly below 1 was expected because potassium ions and hydroxide ions have similar ion radii. The largest increase in specific capacitance obtained by mass balancing was detected for sodium hydroxide. An increase of 8.6% was achieved by shifting the electrode mass ratio from 1 to 0.86; see Table 3. In the case of sodium sulfate and sulfuric acid, increases of 3.7% and 1.5% were obtained. The four curves reveal different curve progressions. The curve corresponding to sodium sulfate, in particular, differs from the other plots. For electrode mass ratios larger than the optimal mass ratio, the specific capacitance declined more slowly than for the other electrolytes. Potassium hydroxide, sodium hydroxide and sulfuric acid produced similar curves.

To eliminate the influence of the electrode thickness or the amount of available active material on the optimal electrode mass ratio, five EDLCs with the optimal electrode mass ratio but varying electrode mass loadings were tested for each electrolyte. The specific capacitance of the correlating EDLCs deviated only slightly, indicating that the influence was negligible.

In Table 3, the theoretical optimal electrode mass ratios are compared with the measured values. The experimental results were approximately consistent with the theoretical optimal electrode mass ratio calculated from the ion size ratio. However, slight deviations due to measurement inaccuracy were found. In the case of sodium sulfate, the theoretical optimal electrode mass ratio differed from the measured ratio to a greater extent.

### Efficiency

**Fig 3:**
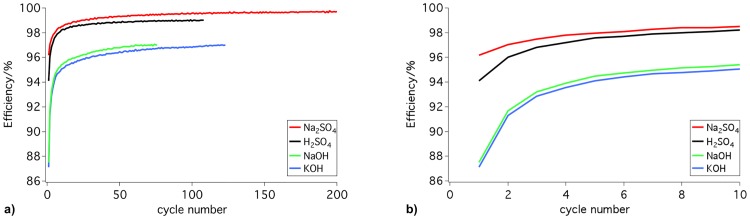
Efficiency of electric double-layer capacitors operating in different electrolytes at the corresponding optimal electrode mass ratio. a) efficiency for each cycle of a 24-hour measurement and b) development of the efficiency during the first 10 cycles of the same measurements.

**Fig 4:**
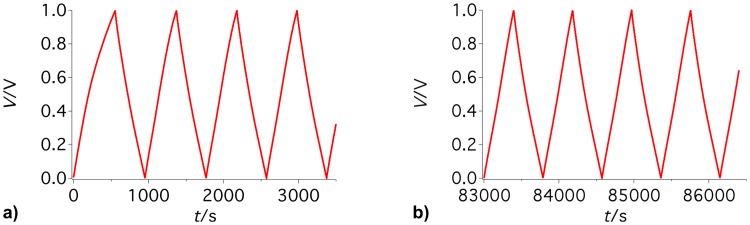
Galvanostatic cycling profile of an electric double-layer capacitor. a) the first cycles and b) the last cycles of a typical 24-hour measurement.

In addition to the capacitance, we also measured the efficiency of the EDLCs from the charge and discharge curves. Fig 3 shows the efficiency of supercapacitors operating with different electrolytes at their optimal electrode mass ratio. All tested EDLCs exhibited a good cycling stability. EDLCs with sulfuric acid or sodium sulfate reached efficiencies of approximately 99%; see Fig 3a. Devices with sodium hydroxide or potassium hydroxide produced slightly lower efficiencies of 97%. After only five charge and discharge cycles, efficiencies of 97–98% for sodium sulfate and sulfuric acid and 94–95% for sodium hydroxide and potassium hydroxide were reached; see Fig 3b. The fast conditioning of the EDLCs can also be observed in Fig 4a and 4b. Fig 4 displays the first and last cycles of a typical 24-hour galvanostatic cycling test. A comparison of the two graphs revealed that the first cycle differed from the following cycles. The curve was bent during charging and discharging. During continuation of the measurements, the curves approached a more linear curve progression. In particular, the fast conditioning of the EDLCs was noteworthy. However, the efficiency was not influenced by the electrode mass ratio.

## Discussion

Although minimal enhancement in specific capacitance was observed for some electrolytes, mass balancing can be a simple approach for increasing the capacitance of EDLCs. For devices using suitable electrolytes, such as sulfuric acid, mass balancing can significantly improve EDLC performance. The mass-balancing approach can also be applied to other electrode-electrolyte combinations and can be implemented in large-scale production processes without any difficulty. Mass balancing further leads to a reduction in unused material and thus a reduction of the weight and volume of EDLCs.

Although one can observe large differences in the specific capacitance between the tested electrolytes, caution should be used when choosing an electrolyte. Sulfuric acid provided the highest specific capacitance in this study, although it might not be the best choice for all supercapacitors. Generally, one should consider the influence of the electrolyte on supercapacitor materials, such as the electrodes, the separator and the passive components. The pH might be crucial in terms of electrode stability, separator stability and corrosion. Neutral electrolytes might be favorable in some supercapacitors. For our system, in which cellulose-based materials were used, neutral electrolytes, e. g. sodium sulfate, are beneficial. However, when acidic or basic electrolytes can be employed, one should use an electrolyte that generates a higher specific capacitance, such as sulfuric acid. Moreover, the performance of the electrolyte is influenced by the electrode material and structure. Thus, recommendations can only be given for particular electrode-electrolyte systems. However, the mass-balancing approach can be applied on all supercapacitors, regardless of the electrode or electrolyte materials used in the system.

To further optimize an EDLC’s performance, one should measure the surface area that is accessible for the anions or cations rather than the electrode mass or the pore size distribution. Narrow pores might only be attainable for small ions. In this case, small ions will have a larger usable surface area than large ions. Having an ion pair with large differences in ion size might result in two different accessible surface areas. Thus, the accessible surface area should be considered to obtain accurate calculations. However, it is not simple to measure the usable surface area for particular ions. Therefore, using the electrode mass to obtain a well-balanced cell is more practical.

Furthermore, one should choose a higher charge and discharge current when performing galvanostatic cycling to obtain faster discharge times that reflect the intended application in KERSs.

Further studies should be conducted to determine the influence of other electrolytes on the optimal electrode mass ratio. The mixture of two or more compatible electrolytes might also be interesting. In addition to aqueous electrolytes, even organic electrolytes and ionic liquids could be investigated.

## Conclusions

In this study, we showed that the capacitance of EDLCs can be increased if the electrode mass ratio is adjusted to the ion size ratio. This optimization is favorable for improving the performance of EDLCs, reducing the amount of unused electrode material and thus decreasing the cell weight, volume and cost. The highest increase in specific capacitance could be achieved for EDLCs using sodium hydroxide as the electrolyte. An increase of 8.6% was obtained by shifting the electrode mass ratio from 1 to 0.86. The highest specific capacitance was obtained with EDLCs using sulfuric acid as the electrolyte. By changing from a symmetric electrode configuration to the optimal electrode mass ratio of 0.75, only a small increase in specific capacitance of 1.5% could be obtained for sulfuric acid.
