# Correction: Electrode Mass Balancing as an Inexpensive and Simple Method to Increase the Capacitance of Electric Double-Layer Capacitors

**DOI:** 10.1371/journal.pone.0170135

**Published:** 2017-01-11

**Authors:** The PLOS ONE Staff

The units of measurement are incorrectly omitted in nine instances throughout the article.

The second sentence of the Materials section should be: Sulfuric acid (analytical grade, from VWR), sodium hydroxide (analytical grade, from VWR), potassium hydroxide (from Fluka) and sodium sulfate (analytical grade, from Merck) were diluted with deionized water to produce electrolytes with a concentration of 1 M.

The second, third, fourth and fifth sentences of the Preparation of TEMPO-oxidized cellulose nanofibers are missing certain units. The correct sentences are: We used 100 g fully bleached softwood Kraft pulp. The pulp was diluted in 10 l deionized water to 2.5% consistency. Then, 2 mmol sodium bromide (NaBr, from Merck Millipore) per g dry pulp and 0.2 mmol/g TEMPO (2,2,6,6-tetramethyl-1-piperidinyloxy, from Sigma-Aldrich) was added to the pulp suspension. The suspension was mixed, and 10 mmol sodium hypochlorite (NaClO, 14%, from VWR) per g dry pulp was added during stirring.

The fourth and seventh sentences of the Preparation of electrodes section are missing certain units. The fourth sentence should be: Approximately 40 ml of deionized water was added to each mixture. The seventh sentence should be: Membrane Filters (filter type: 0.22 μm GV, diameter: 90 mm) using a vacuum filtration funnel. Films with coating weights between 44 g/m^2^ and 154 g/m^2^ were obtained.

The sixth and ninth sentences of the Assembly and testing of electric double-layer capacitors section are missing certain units. The correct sixth sentence is: The EDLCs were cycled for 24 hours between 0 and 1 V with a charge and discharge current of 8 mA. The correct ninth sentence is: This low current resulted in discharge times between 150 and 665 s giving 65 to 288 cycles per 24-hour measurement.
