# Correction: Correction: Electrode Mass Balancing as an Inexpensive and Simple Method to Increase the Capacitance of Electric Double-Layer Capacitors

**DOI:** 10.1371/journal.pone.0174191

**Published:** 2017-03-15

**Authors:** The PLOS ONE Staff

## Notice of Republication

This correction was republished on January 25, 2017, to correct errors that were introduced during the typesetting process. The publisher apologizes for the errors. Please download this article again to view the correct version. The originally published, uncorrected article and the republished, corrected articles are provided here for reference.

## Supporting information

S1 File Originally published, uncorrected article. (PDF)

S2 File Republished, corrected article. (PDF)
